# Beta-Human Chorionic Gonadotropin Producing Osteosarcoma of the Sacrum in a 26-Year-Old Woman: A Case Report and Review of the Literature

**DOI:** 10.1155/2015/897230

**Published:** 2015-02-04

**Authors:** Ryan Glass, Jaya Ruth Asirvatham, Leonard Kahn, Mohamed Aziz

**Affiliations:** Department of Pathology, North Shore Long Island Jewish, New Hyde Park, NY 11040, USA

## Abstract

Ectopic secretion of beta-human chorionic gonadotropin is considered a poor prognostic marker in epithelial tumors. However, very few cases have been reported in sarcomas. We present the case of a 26-year-old female who presented with a metastatic osteosarcoma. She underwent usual testing prior to starting treatment and was found to have elevated levels of beta-human chorionic gonadotropin. As the patient was not pregnant, another source of beta-human chorionic gonadotropin secretion had to be considered. The tumor cells demonstrated positive staining for beta-human chorionic gonadotropin by immunohistochemistry, and serum levels of beta-human chorionic gonadotropin were used to monitor tumor progression and response to chemotherapy. We review the literature and discuss a potential role of beta-human chorionic gonadotropin in the treatment of such patients.

## 1. Introduction

Beta-human chorionic gonadotropin (*β*-hCG) is normally produced by syncytiotrophoblasts of the placenta and may also be secreted by germ cell neoplasms. Ectopic secretion of *β*-hCG by epithelial tumors, including bladder, lung, and colorectal cancers, is a recognized phenomenon associated with a poorer prognosis [1]. However, ectopic *β*-hCG expression has been less frequently reported in sarcomas, with very few known in osteosarcomas. We present a case of a female patient with an osteosarcoma who was incidentally discovered to have elevated serum *β*-hCG levels. Immunohistochemical staining identified the tumor as the source of the hormone and serum levels were used to monitor the tumor's response to treatment and progression.

## 2. Case Report

**Figure 1:**
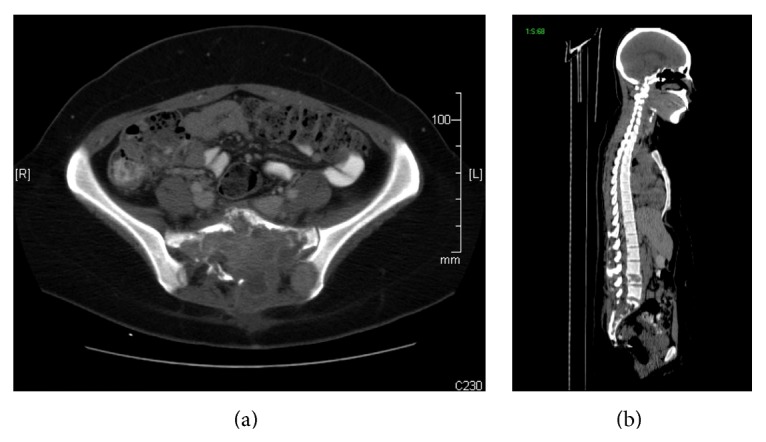
CT Scan showing tumor invasion of sacrum: left and right surrounding parasacral soft tissue (a), and L4 vertebral body (b).

A 26-year-old female presented to an outside institution with pelvic pain over the previous several months. A CT scan revealed a large lytic lesion in the pelvis, multiple paraspinal soft tissue masses, parasacral soft tissue masses, and a lytic lesion in the L4 vertebral body (Figures 1(a) and 1(b)), with a chest X-ray showing multiple lung lesions. Biopsies of the paraspinal masses revealed a high-grade sarcoma. She was seen at our institution approximately one month later. Biopsies taken from the pelvic mass showed identical features to those of the paraspinal masses. During the initial workup, the patient was found to have serum *β*-hCG levels of 693 mIU/mL and pregnancy was suspected. However, pregnancy was ruled out as her menstrual bleeding was 3 weeks earlier and she had never been sexually active. The possibility of a *β*-hCG secreting tumor was considered, and the tumor cells stained strongly positive for *β*-hCG. The final diagnosis was that of a *β*-hCG secreting osteogenic sarcoma. As metastases were present at the time of diagnosis, the patient was not a surgical candidate. Chemotherapy with methotrexate and ifosfamide was initiated. Serum *β*-hCG levels, which were monitored throughout the patient's course, transiently fell after each treatment. This drop correlated with radiologic response to treatment. However, as this was an aggressive tumor, it tended to regrow in spite of treatment. These periods of growth were accompanied by rising *β*-hCG levels.

## 3. Microscopic Description

**Figure 2:**
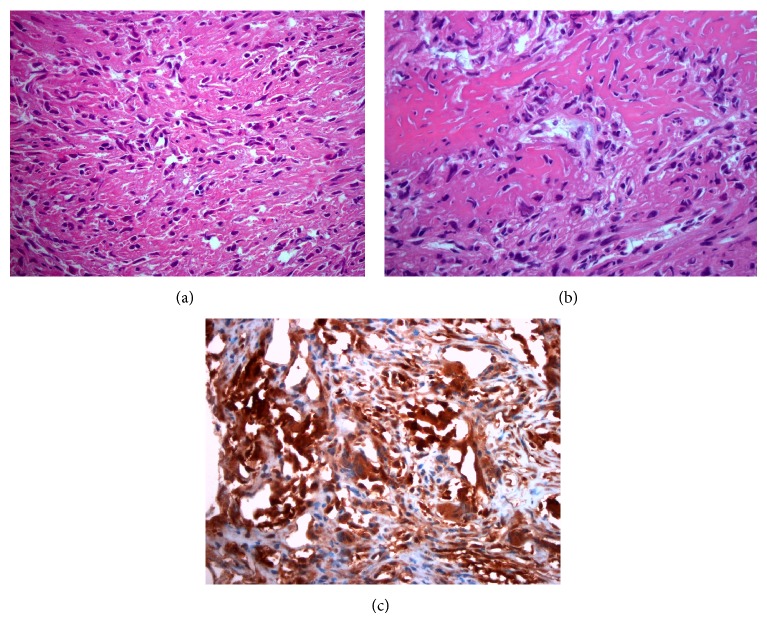
Osteosarcoma of pelvis demonstrating hyperchromatic spindle-shaped tumor cells associated with matrix production (a) and osteoid formation (b). Tumor stains positive for *β*-hCG (c) (Hematoxylin-eosin [a, b], original magnifications ×4 [c], ×20 [a-b]).

Microscopic examination of the biopsy specimens revealed a mass composed primarily of malignant spindle cells, infiltrating bone and soft tissue with no recognizable architectural pattern. The tumor cells appeared cytologically atypical with focal areas of pleomorphism and mitoses up to 4 per 10 high-power fields. Multiple areas of osteoid formation and scattered benign appearing giant cells were noted within the tumor (Figures 2(a) and 2(b)). Initial studies were positive for vimentin, focally positive for CD68, and negative for cytokeratin, CK7, CD34, and S100.

Additional studies were requested after the patient was found to have high levels of *β*-hCG (Figure 2(c)). These revealed the tumor to be positive for *β*-hCG and negative for placental alkaline phosphatase, epithelial membrane antigen, smooth muscle antigen, desmin, myogenin, and CD31.

## 4. Discussion

To the best of our knowledge, very few cases of *β*-hCG secreting osteosarcomas exist in the literature (Table 1). The mean age of these patients was 22 years (range: 5–57 years) with nine females and three males. In cases where serum *β*-hCG testing was performed, levels as high as 5000 mIU/mL have been reported although the significance of this is unclear [2]. Of the ten patients with available follow-up, eight have died or experienced lung metastases. One of Masrouha et al.'s patients, however, experienced a complete response although it should be noted that cells of the initial biopsy stained weakly positive and those from the excised tumor stained negative [5]. Leidinger et al.'s patient also remained disease free, although follow-up was only available for six months [6]. As the majority of these tumors demonstrated an unfavorable rapidly progressing course, this paraneoplastic feature may portend poor prognosis in osteosarcomas.

It is difficult to ascertain the true incidence of *β*-hCG producing osteosarcomas. As most cases in the literature (as well as ours) were discovered incidentally by urine pregnancy screening, it is likely that many others are missed, especially in male patients. To determine the rate of *β*-hCG positivity, Ordonez et al. retrospectively stained 11 osteosarcoma specimens for *β*-hCG, finding that only two (18.2%) stained positive [8]. In a more recent study, by Masrouha et al., 5 of 32 (15.6%) retrospectively stained specimens showed *β*-hCG positivity, 3 of which were from male patients [5].

The significance of *β*-hCG-staining in tumor cells as well is unclear. While it has been shown that staining intensity in carcinomas correlates with tumor grade [1], it is unknown if this relationship holds in osteosarcomas as very few studies have examined this. Leidinger et al. reported positive staining in dedifferentiated metastases of negatively staining primary tumors [6], and Masrouha et al. reported that four of the five cases stained positively in histologically high-grade tumors [5]. However, Oshrine et al. reported no morphological differences between *β*-hCG-positive and *β*-hCG-negative cells within the same tumor [7]. In our case, the tumor appeared of low-grade and, similar to Orhrine's findings, no noticeable differences were seen between *β*-hCG-positive and *β*-hCG-negative areas.

X-ray crystallography of *β*-hCG revealed a quaternary structure containing a “cysteine knot,” making it structurally similar to other members of the “cysteine knot growth factors” (CKGF) super family such as transforming growth factor beta (TGF*β*), platelet derived growth factor (PDGF), vascular endothelial growth factor (VEGF), and bone morphogenic proteins [1]. This finding is consistent with observations linking high serum hCG levels with tumor neovascularization [9], as well as studies showing that addition of hCG may reverse the proapoptotic effects of TGF*β* [10]. hCG may therefore stimulate growth of these tumors through agonistic and antagonistic interactions with receptors of other CKGFs.

Currently, the role of beta-hCG in osteosarcoma management is not well established. However, several prior studies demonstrated that *β*-hCG expressing tumors respond poorly to chemotherapy, and changes in serum levels correlate with relapse and response to treatment [3, 5, 6]. In Boss et al.'s patient, it was found that initial drops followed by increases in *β*-hCG levels corresponded with radiologic response to E7080, an investigational tyrosine kinase inhibitor that targets VEGFR-2 and VEGFR-3 with secondary activity against PDGFR-*β*, FGFR-1, and C-KIT. Taking these observations together, they hypothesized that *β*-hCG could mediate resistance to VEGF inhibition [3], consistent with prior reports of *β*-hCG interactions with CKGF superfamily receptors.

## Figures and Tables

**Table 1:** Previously reported cases of *β*-hCG producing osteosarcoma.

Author	Age	Sex	Chemotherapy	Status
Kalra et al. [2]	21	F	Vinblastine, bleomycin, and cisplatin	Died during treatment

Tuy et al. [3]	37	F	None	Died from disease progression

Boss et al. [4]	57	F	Doxorubicin and cisplatin; ifosfamide and etoposide; Cyclophosphamide and imatinib; E7080	Initial success with E7080, followed by disease progression

Masrouha et al. [5]	17	F	Regimen unknown	Lung metastases
	14	M	Regimen unknown	Died during treatment
	17	F	Regimen unknown	No recurrence
	17	M	Regimen unknown	Lung metastases
	5	M	Regimen unknown	Died during treatment

Leidinger et al. [6]	18	F	COSS-96; COSS-96 with carboplatin, etoposide, and RT	Lost to follow-up

Oshrine et al. [7]	14	F	Doxorubicin and ifosfamide, cisplatin regimen	Disease free after 6 months

Ordonez et al. [8]	26	F	Unknown	Unknown

Our case	26	F	Methotrexate and ifosfamide	Died from progressive disease
